# Constraint-Induced Movement Therapy (CIMT) and Neural Precursor Cell (NPC) Transplantation Synergistically Promote Anatomical and Functional Recovery in a Hypoxic-Ischemic Mouse Model

**DOI:** 10.3390/ijms25179403

**Published:** 2024-08-29

**Authors:** Prakasham Rumajogee, Svetlana Altamentova, Junyi Li, Nirushan Puvanenthirarajah, Jian Wang, Azam Asgarihafshejani, Derek Van Der Kooy, Michael G. Fehlings

**Affiliations:** 1 Division of Genetics and Development, Krembil Brain Institute, University Health Network, Toronto, ON M5T 2S8, Canada; prakasham.rumajogee@googlemail.com (P.R.); azam.asgarihafshejani@uhn.ca (A.A.); 2 Institute of Medical Science, University of Toronto, Toronto, ON M5S 3E1, Canada; derek.van.der.kooy@utoronto.ca; 3 Division of Neurosurgery and Spine Program, Department of Surgery, University of Toronto, Toronto, ON M5T 1P5, Canada

**Keywords:** cerebral palsy, neonatal stroke, hypoxia-ischemia, oligodendrocyte, myelination, white matter injury, neural precursor cells, constraint-induced movement therapy, botox, rehabilitation

## Abstract

Cerebral palsy (CP) is a common neurodevelopmental disorder characterized by pronounced motor dysfunction and resulting in physical disability. Neural precursor cells (NPCs) have shown therapeutic promise in mouse models of hypoxic-ischemic (HI) perinatal brain injury, which mirror hemiplegic CP. Constraint-induced movement therapy (CIMT) enhances the functional use of the impaired limb and has emerged as a beneficial intervention for hemiplegic CP. However, the precise mechanisms and optimal application of CIMT remain poorly understood. The potential synergy between a regenerative approach using NPCs and a rehabilitation strategy using CIMT has not been explored. We employed the Rice–Vannucci HI model on C57Bl/6 mice at postnatal day (PND) 7, effectively replicating the clinical and neuroanatomical characteristics of hemiplegic CP. NPCs were transplanted in the corpus callosum (CC) at PND21, which is the age corresponding to a 2-year-old child from a developmental perspective and until which CP is often not formally diagnosed, followed or not by Botulinum toxin injections in the unaffected forelimb muscles at PND23, 26, 29 and 32 to apply CIMT. Both interventions led to enhanced CC myelination and significant functional recovery (as shown by rearing and gait analysis testing), through the recruitment of endogenous oligodendrocytes. The combinatorial treatment indicated a synergistic effect, as shown by newly recruited oligodendrocytes and functional recovery. This work demonstrates the mechanistic effects of CIMT and NPC transplantation and advocates for their combined therapeutic potential in addressing hemiplegic CP.

## 1. Introduction

Cerebral palsy (CP), affecting 2–3.5/1000 births, encompasses a spectrum of conditions resulting in non-progressive developmental brain injury [1,2]. CP presents with motor dysfunctions, frequently accompanied by sensory alterations, cognitive deficits, epilepsy, and secondary musculoskeletal complications [3]. The economic burden of CP is profound, costing approximately one million CAD per patient and, with an estimated global prevalence of 17 million affected individuals, the societal as well as healthcare implications are substantial. Current therapeutic avenues for CP are limited and include therapeutic hypothermia [4], surgical interventions addressing secondary complications [1], and rehabilitative methodologies [5].

Constraint-induced movement therapy (CIMT) has been employed clinically to enhance functional recovery of the hemiparetic upper extremity post-stroke in adults [6,7] and in pediatric patients following perinatal stroke with hemiplegia [8,9,10]. Pediatric CIMT is now a therapeutic strategy for managing children with hemiplegic CP [11,12,13,14,15,16]. This rehabilitative approach encompasses (i) constraining the unaffected upper extremity; (ii) intensive, repetitive motor training of the affected limb; and (iii) targeted training with positive reinforcement (shaping), focusing on more complex motor activities [10]. Although CIMT’s clinical efficacy is evident, the underlying neurobiological mechanisms remain elusive. Prevailing hypotheses suggest involvement of brain plasticity, particularly in cortico-motor circuitry reorganization [10,17]. Recent animal research corroborates CIMT’s efficacy in models of adult stroke [18,19,20,21,22,23] and neonatal brain injury [24,25,26,27].

Current treatments have demonstrated partial functional improvements in CP patients [28], but no cure currently exists due to a lack of therapies capable of repairing the injured WM [5,29]. Stem cell therapy, particularly neural precursor cell (NPC) transplantation, has emerged as a promising regenerative avenue. In our recent study [30], we optimized a neonatal hypoxic-ischemic (HI) hemiplegic mouse model based on the Rice–Vannucci model [31]. We demonstrated positive outcomes after NPC transplantation in the corpus callosum (CC) via a trophic effect leading to the restoration of various brain structures, remyelination of the CC, and functional recovery [30]. In CP, the WM is a primary target which, after perinatal injury, fails to myelinate properly. For instance, a relationship has been demonstrated between CC integrity and upper-extremity function in children with spastic CP [32]. Likewise, impaired myelination is a central feature of the HI model [2,30,33].

In this study, using our HI model [30], we optimized a CIMT protocol based on intramuscular injections of botulinum toxin in the uninjured forelimb to paralyze it and to promote the use of the impaired forelimb. Extending our prior findings, we evaluated the individual and combined effects of CIMT (rehabilitation) and NPC transplantation (regeneration). We used a range of techniques (e.g., histology, immunohistochemistry, electrophysiology, and behavioural testing) to explore the potential of CIMT to restore areas of WM loss and neurobehavioural function. This study further clarifies the underlying mechanisms of NPC transplantation as well as CIMT and, for the first time, demonstrates a promising synergistic effect of these two therapeutic modalities for the treatment of hemiplegic CP.

## 2. Results

**Figure 1:**
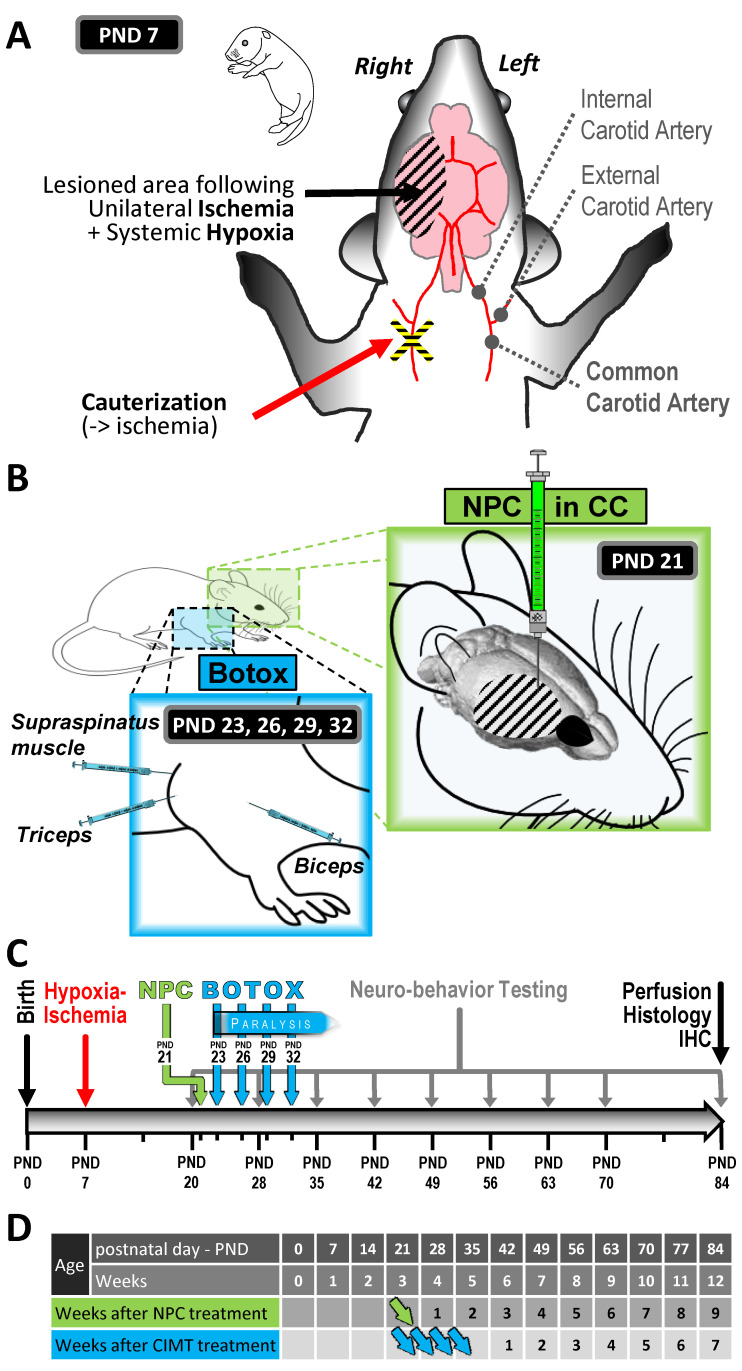
Experimental design and timeline. HI injury at PND7 included ischemia (i.e., right common carotid artery cauterization) and hypoxia (8% O_2_ for 45 min) (**A**). The regeneration strategy was based on NPC transplantation in the right/injured corpus callosum at PND21 (developmentally comparable to a 2-year old child, the age until which CP is not formally diagnosed) and the rehabilitation strategy was based on Botox injections into the biceps, triceps, and supraspinous muscles of the right/unaffected forelimb at PND23, 25, 29, and 32 to reproduce CIMT (**B**). Timeline of the experimental procedures in neurobehavioural testing, the PND77 time-point was skipped as the analysis of the partial data obtained at PND70 showed a “plateau” of the values, and consequently, a time-point after two weeks (PND84 following PND70) was sought (**C**). Time-points of the experimental procedures (**D**). The body weights of animals under CIMT or CIMT + NPC were comparable to the control body weights (see Supplemental Figure S1). Green arrow represents the NPC transplantation. Blue arrows represent the Botox injections.

All experiments were conducted according to the timeline described in Figure 1.

### 2.1. Transplanted NPCs Survive, Migrate, Differentiate Morphologically, and Integrate in the Corpus Callosum

**Figure 2:**
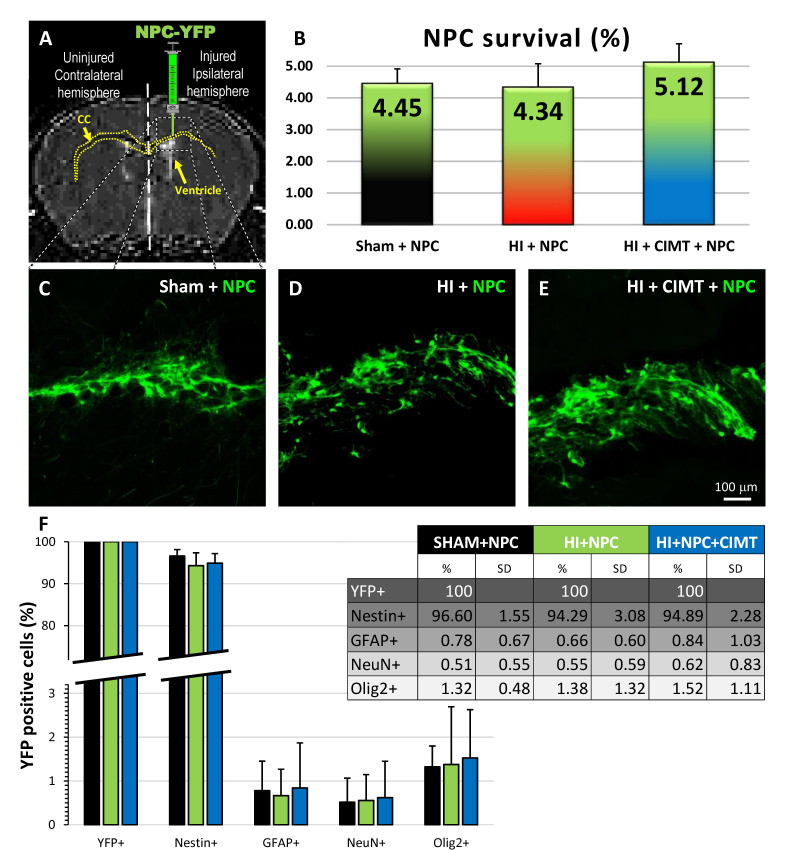
Transplanted NPCs survive, migrate, differentiate morphologically, and integrate in the corpus callosum (CC). MRI picture showing the location of transplantation of YFP-NPCs, i.e., the right/injured CC, ipsilateral to the common carotid occlusion (**A**). Survival rate of transplanted NPCs in the sham, HI, and HI + CIMT mice (**B**). Transplanted NPCs migrate, differentiate morphologically, and integrate in the brain tissue of the sham (**C**), HI (**D**), and HI + CIMT mice (**E**). The vast majority of transplanted NPCs remain Nestin-positive (**F**).

NPCs were transplanted at PND21 into the injured CC (Figure 2A). After 9 weeks (PND84), the NPC survival rate (based on the total number of NPCs transplanted and the number of surviving cells in the brain (YFP+) that was estimated using StereoInvestigator^®^) was comparable after transplantation in the sham group (4.45 ± 0.46%, one-way ANOVA, followed by Bonferroni’s test, F = 2.34), to the rate in HI (4.34 ± 0.74%, *p* > 0.9999, t = 0.28) and in HI + CIMT (5.12 ± 0.59%, *p* = 0.4146, t = 1.59) animals (Figure 2B). Also, there was no difference after transplantation in the HI and HI + CIMT animals (*p* = 0.1854, t = 2.06). After transplantation, NPCs survived, migrated through the CC, colonized the injury site, and engrafted in the brain tissue. The initial shape progressively changed from a round-type cell in suspension to a multipolar shape, as the NPCs grew numerous processes into the neighboring brain structures and established a network 9 weeks after treatment, as shown in Figure 2C–E. This suggests the establishment of some level of communication with the surrounding brain structures, such as the hippocampus, the cortex, and the internal capsule. We observed, as shown in our previous publication, that YFP + NPCs are present up to 19 weeks after treatment [30]. Prior to transplantation, the NPCs (YFP+) grown in vitro were immunopositive for Nestin. The majority were still expressing Nestin 9 weeks after transplantation in the CC of the sham group (96.60 ± 1.55%; Kruskal–Wallis Test; *p* = 0.123), HI (94.29 ± 3.08%; Kruskal–Wallis Test; *p* = 0.210), and HI + CIMT (94.89 ± 2.28%; Kruskal–Wallis Test; *p* = 0.198) mice. When transplanted in the sham, HI, or HI + CIMT mice, very few NPCs differentiated into astrocytes (respectively 0.78 ± 0.67%, 0.66 ± 0.60% and 0.84 ± 1.03%), neurons (0.51 ± 0.55%, 0.55 ± 0.59% and 0.62 ± 0.83%), and oligodendrocytes (1.32 ± 0.48%, 1.38 ± 1.32% and 1.52 ± 1.11%) (Figure 2F).

### 2.2. Transplanted NPCs, CIMT, and NPC + CIMT Lead to Structural Recovery

**Figure 3:**
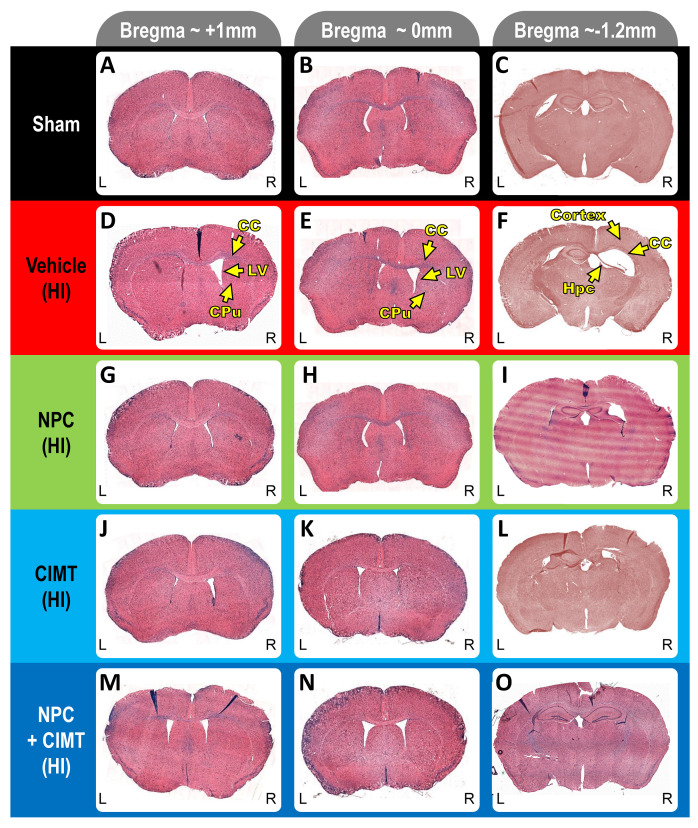
The treatments significantly improved various brain structures following HI injury. Representative pictures of LFB/H&E staining of the whole brain coronal sections at 3 Bregma levels (i.e., Bregma −1.2, 0 and +1 mm) for the sham (**A**–**C**), HI (**D**–**F**), HI + NPC (**G**–**I**), HI + CIMT (**J**–**L**), and HI + NPC + CIMT (**M**–**O**) mice. Yellow arrows indicate brain structures such as the CC, lateral ventricle (LV), caudate putamen (CPu), hippocampus (Hpc), and cortex. L and R indicate the left- and right-hand sides of the brain, respectively.

Following neonatal HI, unilateral lesions occurred in the right ipsilateral brain hemisphere. Figure 3 shows representative pictures of coronal sections at 3 Bregma levels (approximatively −1.2, 0, and +1 mm). Different structures such as the CC, cortex, hippocampus, lateral ventricle, or caudate putamen are affected after HI (Figure 3D–F, arrows) as compared to the sham group (Figure 3A–C). Substantial levels of recovery were observed after NPC transplantation (Figure 3G–I, NPC), CIMT (Figure 3J–L), and NPC + CIMT (Figure 3M–O).

#### 2.2.1. Neonatal HI Reduces the Overall Brain Size, but No Treatments Restore It

**Figure 4:**
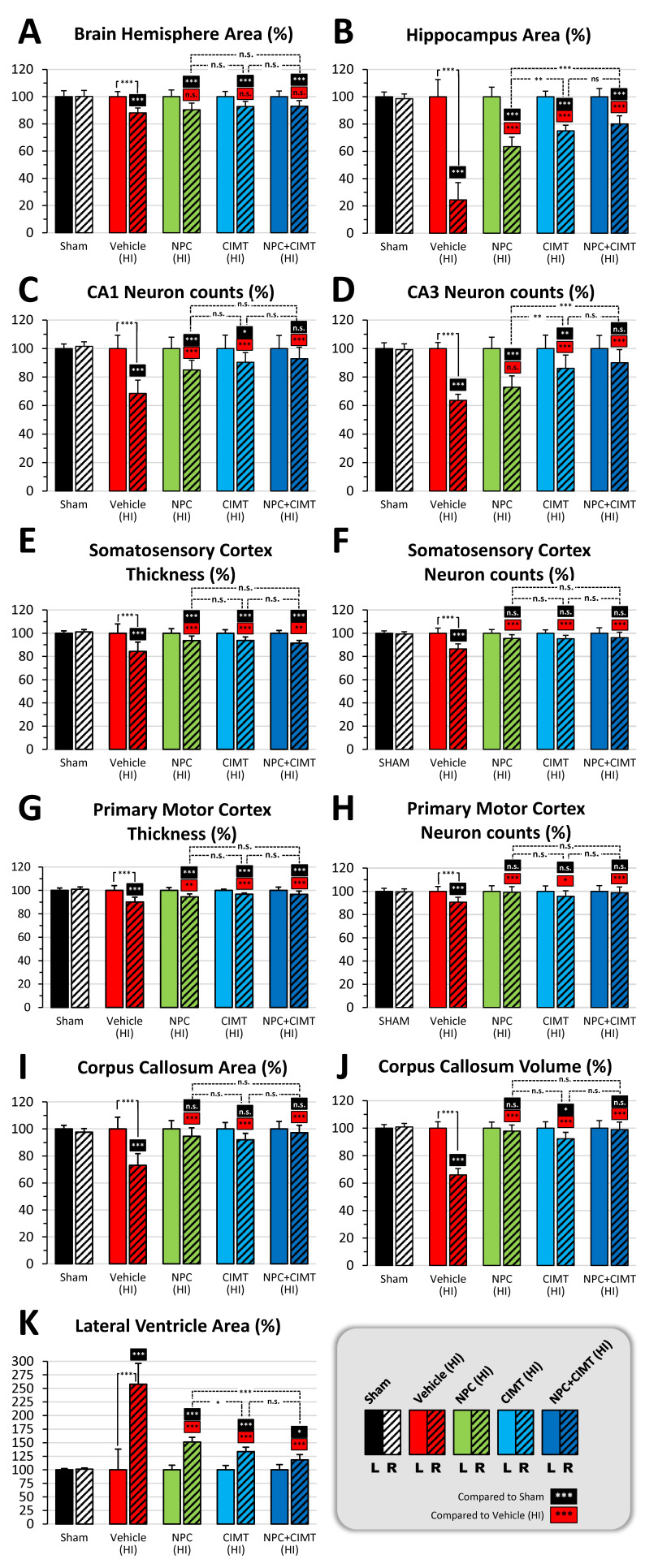
Assessment of structural integrity and neuronal populations in various brain structures following NPC, CIMT, and NPC + CIMT treatments. Following HI, various brain structures were reduced in size and displayed a decreased number of neurons. The brain hemisphere area decrease was not restored following any of the treatments (**A**). Even though the reduced hippocampus area was not fully restored, it was significantly improved when compared to the sham group. CIMT and NPC + CIMT treatments led to better recovery than NPCs used alone (**B**). The CA1 neuronal population significantly improved after treatments but was fully restored only following NPC + CIMT treatment (**C**). The CA3 neuronal population significantly improved after CIMT and NPC + CIMT, but not NPC treatment, and was fully restored after NPC + CIMT treatment (**D**). The somatosensory cortex thickness was partially restored after all treatments (**E**), while the neuronal population increased only slightly (**F**). The primary motor cortex thickness was partially restored after all treatments (**G**) while the neuronal population slightly increased (**H**). The CC area (**I**) and volume (**J**) were fully restored after all treatments. The lateral ventricle area significantly improved, but was not fully restored, after any treatments. However, NPC + CIMT led to better recovery than both treatments alone (**K**). The % sign indicates that the left side was normalized to 100% and compared with the right side. The dashed versus plain bars represent the right versus left side. * *p* < 0.05, ** *p* < 0.01, *** *p* < 0.001, n.s.—no significance.

After HI, the size of the right injured brain hemisphere was significantly reduced (88.05 ± 3.66%, two-way ANOVA, followed by Bonferroni’s test, F = 7.67) as compared to both the uninjured contralateral hemisphere (normalized at 100, *p* < 0.0001, t = 6.91) and the ipsilateral hemisphere of the sham animals (100.14 ± 4.39%, *p* < 0.0001, t = 7.13) (Figure 4A). The size of the right brain hemisphere remained smaller after NPC transplantation (90.36 ± 4.86%, *p* > 0.9999, t = 1.52), CIMT (92.78 ± 3.81%, *p* = 0.0982, t = 3.11), and NPC + CIMT (92.92 ± 4.16%, *p* = 0.0694, t = 3.22) compared to the vehicle (88.05 ± 3.66%) (Figure 4A). Therefore, the three treatments do not have a clear impact on the overall brain size.

#### 2.2.2. HI Injury Significantly Affects the Hippocampus, and Treatments Mitigate the Damage

In HI injured animals, the right hippocampus was dramatically smaller (24.48 ± 12.57%, two-way ANOVA, followed by Bonferroni’s test, F = 95.26) as compared to the uninjured contralateral hemisphere (normalized at 100, *p* < 0.0001, t = 27.61) or the ipsilateral hemisphere of the sham mice (98.51 ± 3.47%, *p* < 0.0001, t = 25.52) (Figure 4B).

The right hippocampus size significantly increased after NPC transplantation in the CC of HI mice (63.45 ± 6.96%, *p* < 0.0001, t = 14.24), CIMT (75.05 ± 4.11%, *p* < 0.0001, t = 18.49), and NPC + CIMT (80.10 ± 5.97%, *p* < 0.0001, t = 20.33), when compared to the vehicle animals (HI injured, 24.48 ± 12.57%). However, the recovery was not optimal when compared to the sham ipsilateral group (98.51 ± 3.47%) for NPC (*p* < 0.0001, t = 12.09), CIMT (*p* < 0.0001, t = 8.09), and NPC + CIMT (*p* < 0.0001, t = 6.35). While all three treatments led to recovery, better results were obtained after CIMT (*p* = 0.0018, t = 4.24) and NPC + CIMT (*p* < 0.0001, t = 6.09) treatments than NPC treatment alone (Figure 4B).

**Figure 5:**
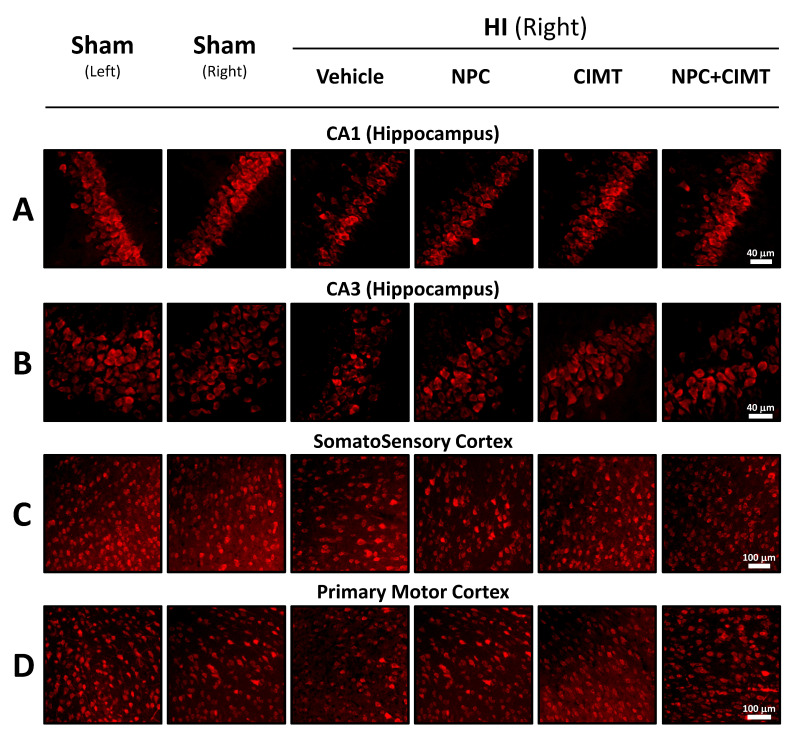
Pictures of fluorescent NeuN-positive cells in the hippocampus and the cortex following NPC, CIMT, and NPC + CIMT treatments. Following HI, the neuronal population was reduced in various brain structures such as the CA1 (**A**) and CA3 (**B**) of the hippocampus, as well as the somatosensory (**C**) and primary motor cortex (**D**).

The NeuN+ population was dramatically reduced following HI injury in the ipsilateral CA1 region (68.55 ± 9.32%, ANOVA, followed by Bonferroni’s test, F = 12.58) as compared to the uninjured contralateral CA1 (normalized at 100, *p* < 0.0001, t = 8.99) or to the ipsilateral CA1 of the sham animals (101.54 ± 3.17%, *p* < 0.0001, t = 9.44) (Figure 4C and Figure 5A).

A significant increase to the neuronal population in the ipsilateral CA1 was observed after NPC transplantation (84.94 ± 6.88%, *p* = 0.0002, t = 4.80), CIMT (90.32 ± 6.88%, *p* < 0.0001, t = 6.82), and NPC + CIMT (92.87 ± 8.05%, *p* < 0.0001, t = 7.62) when compared to vehicle animals (68.55 ± 9.32%). However, the recovery was limited when compared to the sham ipsilateral group (101.54 ± 3.17%) for NPC (*p* = 0.0002, t = 4.86) and CIMT (*p* = 0.0286, t = 3.51), unlike for NPC + CIMT (*p* = 0.3455, t = 2.715). All three treatments led to significant but partial recovery, with no difference between them in terms of the CA1 neuronal population (Figure 4C and Figure 5A).

Similarly, the CA3 NeuN+ population was dramatically reduced after HI injury in the ipsilateral hippocampus (63.66 ± 4.20%, ANOVA, followed by Bonferroni’s test, F = 17.51) as compared to the uninjured contralateral CA3 (normalized at 100, *p* < 0.0001, t = 10.49) or to the ipsilateral CA3 of the sham animals (99.30 ± 3.97%, *p* < 0.0001, t = 10.29) (Figure 4D and Figure 5B).

A significant increase to the neuronal population in the ipsilateral CA3 was observed after CIMT (86.08 ± 9.38%, *p* < 0.0001, t = 7.09) and NPC + CIMT (90.05 ± 9.22%, *p* < 0.0001, t = 8.34), but not after NPC transplantation (72.90 ± 7.98%, *p* = 0.3309, t = 2.73), when compared to vehicle animals (63.66 ± 4.20%). The recovery was limited when compared to the sham ipsilateral group (99.30 ± 3.97%) for NPC (*p* < 0.0001, t = 7.80) and CIMT (*p* = 0.0026, t = 4.18), unlike for NPC + CIMT (*p* = 0.1880, t = 2.92). Therefore, all three treatments led to a substantial, although not full, recovery, with no difference between them in terms of the CA3 neuronal population (Figure 4D and Figure 5B).

#### 2.2.3. HI Injury Affects the Cortex, and Treatment Produces Significant Recovery

After HI, the ipsilateral somatosensory cortex (SSC) became thinner (84.35 ± 8.00%, two-way ANOVA, followed by Bonferroni’s test, F = 18.95) compared to the contralateral side (normalized at 100, *p* < 0.0001, t = 9.25) or to the ipsilateral side of the sham animals (101.10 ± 2.03%, *p* < 0.0001, t = 11.73) (Figure 4E).

A slight increase in SSC thickness occurred after NPC transplantation (93.64 ± 3.86%, *p* = 0.0001, t = 4.86), CIMT (93.78 ± 3.05%, *p* < 0.0001, t = 5.28), and NPC + CIMT (91.48 ± 2.32%, *p* < 0.0001, t = 6.95), when compared to the vehicle animals (84.35 ± 8.00%), but did not reach significance (Figure 4E).

A significant decrease in the neuronal population was observed after HI injury in the ipsilateral SSC (86.47 ± 4.41%, two-way ANOVA, followed by Bonferroni’s test, F = 10.08) as compared to the contralateral control side (normalized at 100, *p* < 0.0001, t = 9.38) or the ipsilateral side of the sham animals (99.29 ± 1.99%, *p* < 0.0001, t = 7.75) (Figure 4F and Figure 5C). Neuronal populations of the SSC significantly increased after NPC transplantation (95.51 ± 3.21%, *p* < 0.0001, t = 6.58), CIMT (95.29 ± 2.84%, *p* < 0.0001, t = 6.42), and NPC + CIMT (96.18 ± 4.69%, *p* < 0.0001, t = 7.07) (Figure 4F and Figure 5C).

Similarly, the ipsilateral primary motor cortex (PMC) was thinner after HI (90.07 ± 4.14%, two-way ANOVA, followed by Bonferroni’s test, F = 18.78) compared to the contralateral side (normalized at 100, *p* < 0.0001, t = 9.12) or to the ipsilateral side of the sham animals (100.89 ± 2.07%, *p* < 0.0001, t = 11.78) (Figure 4G).

A slight increase to the PMC thickness occurred after NPC transplantation (94.50 ± 2.48%, *p* = 0.008, t = 3.86), CIMT (96.76 ± 1.03%, *p* < 0.0001, t = 6.28), and NPC + CIMT (91.48 ± 2.32%, *p* < 0.0001, t = 6.21), when compared to the vehicle animals (90.07 ± 4.14%) (Figure 4G).

As for the neuronal population in the SSC, the NeuN+ cell population significantly decreased in the HI injured ipsilateral PMC (90.75 ± 4.16%, two-way ANOVA, followed by Bonferroni’s test, F = 6.613) as compared to the contralateral control side (normalized at 100, *p* < 0.0001, t = 6.44) or the ipsilateral side of the sham animals (99.57 ± 2.59%, *p* < 0.0001, t = 5.36) (Figure 4H and Figure 5D). Neuronal populations of the PMC significantly increased after NPC transplantation (99.19 ± 4.84%, *p* < 0.0001, t = 6.18), CIMT (95.81 ± 4.71%, *p* = 0.0144, t = 3.70), and NPC + CIMT (98.80 ± 4.95%, *p* < 0.0001, t = 5.89) (Figure 4H and Figure 5D).

#### 2.2.4. HI Injury Significantly Affects the Corpus Callosum, and Treatment with NPCs and CIMT Leads to Recovery

The analysis of the anterior and posterior coronal sections (Figure 4I) showed a significant decrease in the right injured CC area (73.07 ± 8.70%, two-way ANOVA, followed by Bonferroni’s test, F = 23.42) compared to the uninjured contralateral CC (normalized at 100, *p* < 0.0001, t = 12.76) or to the ipsilateral CC of the control animals (97.56 ± 2.64%, *p* < 0.0001, t = 10.47).

A complete restoration of the CC area was obtained after NPC transplantation (94.60 ± 6.15%, *p* = 0.0001, t = 10.87), CIMT (92.01 ± 4.77%, *p* < 0.0001, t = 9.46), and NPC + CIMT (97.20 ± 5.44%, *p* < 0.0001, t = 12.05) when compared to the vehicle animals (73.07 ± 8.70%). In addition, the CC area became similar to the control volume (ipsilateral CC of the sham group: 97.56 ± 2.64%) after NPC (*p* > 0.9999, t = 1.33), CIMT (*p* = 0,6408, t = 2.48), and NPC + CIMT (>0.9999, t = 0.16) transplantation (Figure 4I).

The CC volumetric assessment (Figure 4J) at the level of the transplantation site (i.e., from Bregma −1.5 mm to +1.5 mm) showed a significant decrease in the right injured CC volume (66.03 ± 4.66%, two-way ANOVA, followed by Bonferroni’s test, F = 39.29) compared to the uninjured contralateral CC (normalized at 100, *p* < 0.0001, t = 15.13) or to the ipsilateral CC of the sham animals (100.85 ± 2.57% *p* < 0.0001, t = 14.36).

The volume of the right CC was restored after NPC transplantation (97.89 ± 4.40%, *p* < 0.0001, t = 13.70), CIMT (92.38 ± 4.59%, *p* < 0.0001, t = 11.73), and NPC + CIMT (98.94 ± 5.43%, *p* < 0.0001, t = 14.16), when compared to the vehicle (66.03 ± 4.66%). These volumes became similar to the control volumes (ipsilateral CC of the sham group: 100.85 ± 2.57%) after NPC (*p* > 0.9999, t = 1.19), CIMT (*p* = 0,0399, t = 3.39), and NPC + CIMT (>0.9999, t = 0.77). The corpus callosum was fully recovered after NPC transplantation.

#### 2.2.5. HI Injury Notably Affects the Lateral Ventricle, and Treatment with NPCs and CIMT Leads to Significant Recovery

The histological analysis demonstrated clear unilateral damage to various structures in the right brain hemisphere following HI insult, including a significant enlargement of the right lateral ventricle (LV) (Figure 3, D,E versus A,B, yellow arrow). Area measurements (two-way ANOVA, followed by Bonferroni’s test, F = 100.7) showed that the right LV was dramatically enlarged (257.80 ± 38.20%) compared to the contralateral LV (normalized at 100, *p* < 0.0001, t = 25.73) and to the ipsilateral LV of the sham animals (101.13 ± 2.30%, *p* < 0.0001, t = 23.65) (Figure 4K).

The right LV size was partially restored after NPC transplantation (151.32 ± 8.55%, *p* < 0.0001, t = 18.68) and CIMT (133.64 ± 8.06%, *p* < 0.0001, t = 21.79), but was almost completely restored after NPC + CIMT (116.43 ± 9.58%, *p* < 0.0001, t = 24.46), when compared to the vehicle group (257.80 ± 38.20%). When compared to the NPC-treated animals (151.32 ± 8.55%), NPC + CIMT was more efficient (116.43 ± 9.58%, *p* < 0.0001, t = 6.289) as well as, to a lesser extent, CIMT (133.64 ± 8.06%, *p* = 0,0410, t = 3.38) (Figure 4K).

### 2.3. Transplanted NPCs, CIMT, and the Combination of These Modalities Leads to Myelination

#### 2.3.1. Following HI, Treatment with NPCs and CIMT Restored the Endogenous OL Population above the Normal Level, While the Mature OL Population Was Normalized

**Figure 6:**
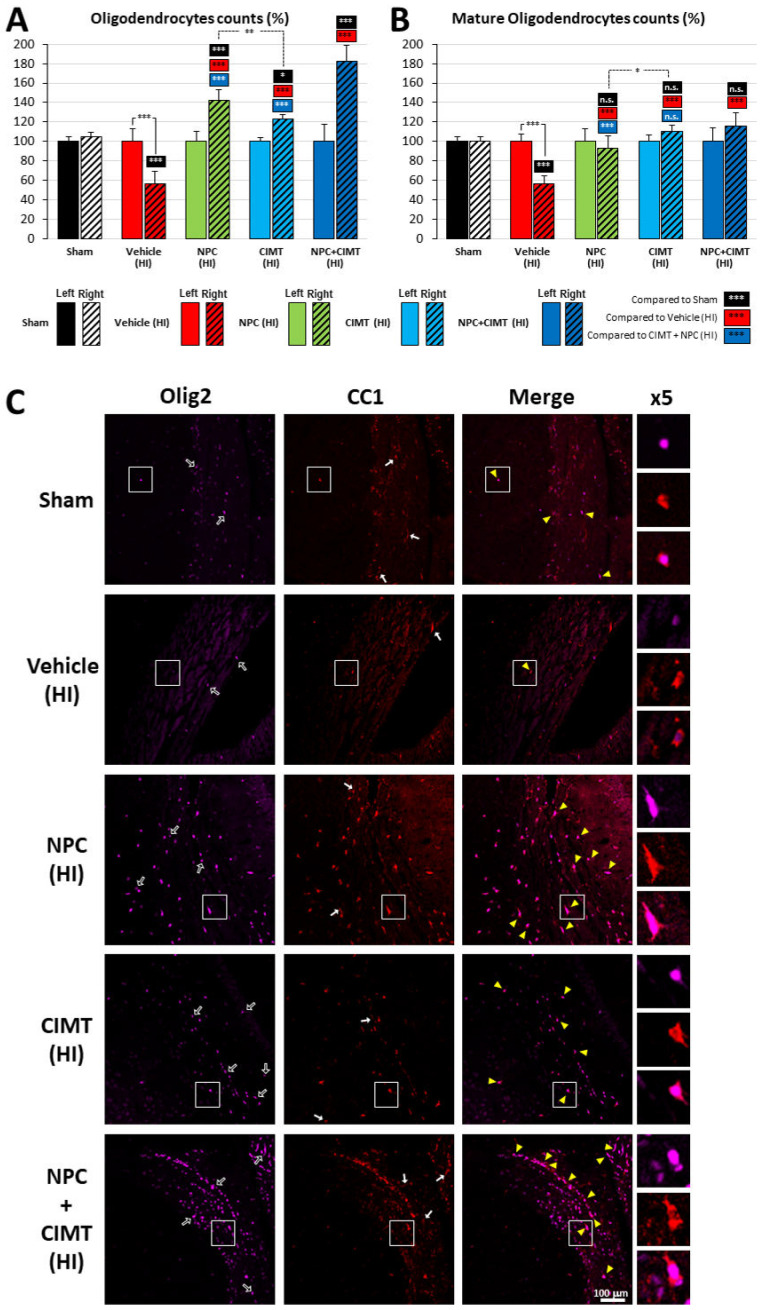
Effect of the treatments on the oligodendrocyte population in the corpus callosum. Following HI, the oligodendrocyte population (Olig2+ cells) was dramatically reduced in the ipsilateral brain (red dashed bar) when compared to the contralateral brain (control side, red plain bar). After all treatments, the OL population increased above normal levels. Following the NPC + CIMT treatment, this increase was significantly higher than after both treatments alone (**A**). The mature oligodendrocyte population (Olig2+/CC1+ cells) returned to normal levels after all treatments (**B**). Representative pictures of immunostaining for oligodendrocytes (Olig2+/CC1−; black arrows), astrocytes (Olig2−/CC1+; white arrows), and mature oligodendrocytes (CC1+/Olig2+; yellow arrowheads) in the CC of the sham and HI animals after treatments (NPC, CIMT, and NPC + CIMT). The white squares indicate an example of mature oligodendrocyte enlarged five times (**C**). The left side was normalized to 100% and compared with the right side. The dashed versus plain bars represent the right versus left side. * *p* < 0.05, ** *p* < 0.01, *** *p* < 0.001, n.s.—no significance.

Following HI insult, the endogenous OL (Olig2+) population was significantly decreased in the injured right CC (55.97 ± 13.14%, two-way ANOVA, followed by Bonferroni’s test, F = 57.25) compared to the uninjured contralateral CC (normalized at 100, *p* < 0.0001, t = 6.689) or the ipsilateral CC of the sham animals (104.52 ± 4.84%, *p* < 0.0001, t = 7.654) (Figure 6A).

The OL population was completely restored and even went above the control level (i.e., 100%) after NPC transplantation (142.69 ± 10.69%, *p* = 0.0001, t = 15.21), CIMT (123.09 ± 4.27%, *p* < 0.0001, t = 11.17), and NPC + CIMT (182.04 ± 17.20%, *p* < 0.0001, t = 21.41), when compared to the vehicle (55.97 ± 13.14%). The OL population following CIMT (123.09 ± 4.27%) was lower than the OL population obtained after NPC treatment (142.69 ± 10.69%, *p* = 0.0092, t = 3.90). However, the level attained after NPC + CIMT (NPC + CIMT: 182.04 ± 17.20%) was significantly higher than the one achieved after both treatments alone (NPC: 142.69 ± 10.69%, *p* < 0.0001, t = 8.06; CIMT: 123.09 ± 4.27%, *p* < 0.0001, t = 11.25) (Figure 6A).

However, the vast majority of those Olig2+ cells were also negative for the YFP-tag applied to transplanted NPCs, confirming their endogenous origin. As demonstrated in our recent publication [30] and confirmed here, the majority of transplanted NPCs remained positive for the Nestin marker regardless of the treatment (Figure 2F).

The mature OL population (CC1+/Olig2+) (Figure 6B) was also affected by HI injury (two-way ANOVA, followed by Bonferroni’s test, F = 16.19). A decrease was observed (56.77 ± 7.45%) as compared to the uninjured contralateral CC (normalized at 100, *p* < 0.0001, t = 7.00) and the ipsilateral CC of the control animals (99.82 ± 4.71%, *p* < 0.0001, t = 7.23).

The mature OL population was restored after NPC (93.14 ± 13.00%, *p* > 0.9999, t = 1.31), CIMT (109.91 ± 6.83%, *p* > 0.9999, t = 1.87), and NPC + CIMT (115.44 ± 14.22%, *p* = 0.1817, t = 2.96) treatments and was returned to normal when compared to the sham group (99.82 ± 4.71%).

Figure 6C shows representative immunofluorescence images of the CC tissue processed with Olig2 and CC1 markers: Olig2+/CC1− stained immature OLs (black arrows), while Olig2−/CC1+ revealed astrocytes (white arrows) and Olig2+/CC1+ marked mature OLs (yellow arrowheads). After HI (row 2), a significant decrease in the OL population (including mature forms) was observed. The three treatments (i.e., NPC in row 3, CIMT in row 4, and NPC + CIMT in row 5), to various extents, restored these cell populations.

#### 2.3.2. HI Injury Significantly Affects Myelination of the CC and Treatment with NPCs and CIMT Leads to Functional Myelination

**Figure 7:**
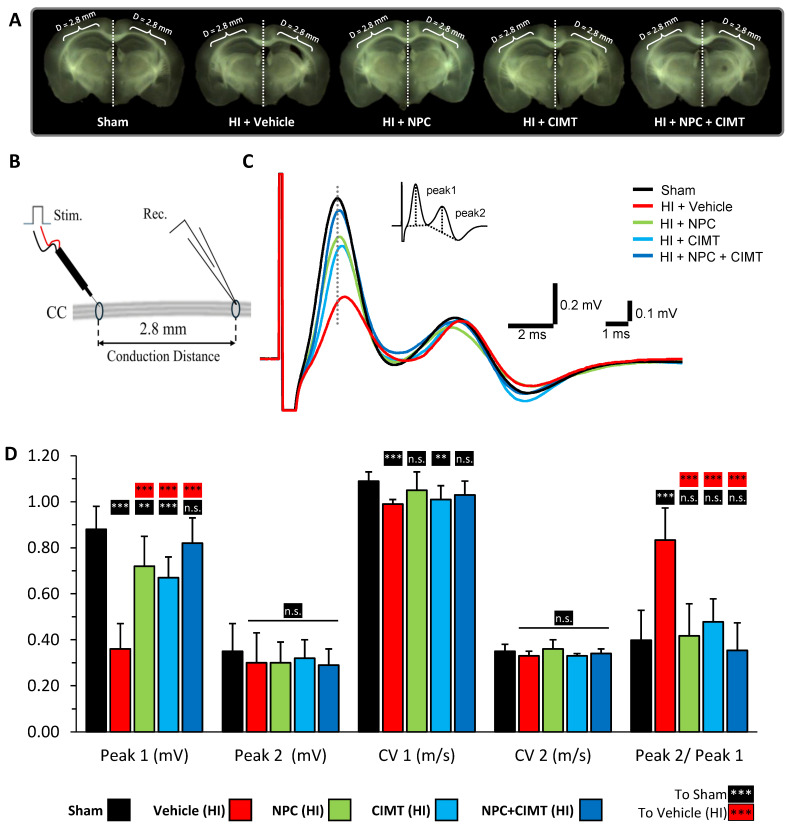
All treatments led to functional myelination, with an enhanced synergistic effect observed with the combinatorial treatment. Representative pictures of coronal brain sections prepared for electrophysiology (**A**). Diagram of the suction electrode recording method for the CC with a conduction distance of 2.8 mm (**B**). Averaged compound action potential (CAP) recordings from the right/injured side of the CC of the sham animals (black line); HI injured animals (red line); and HI injured animals treated with NPCs (green line), CIMT (blue line), and NPC + CIMT (dark-blue line) ((**C**) inset: the measurement of CAP amplitudes). Statistical assessment of Peak 1 and Peak 2 amplitude (mV), conduction velocity CV1 and CV2 (m/s), and Peak 2/Peak 1 ratio amplitude in the right/injured side of the CC compared to the left/control side of the CC for all studied animal groups: sham animals (black bar, *n* = 21); HI injured animals (red bar, *n* = 9); HI injured animals treated with NPC (green bar, *n* = 8), CIMT (blue bar, *n* = 8), and NPC + CIMT (dark-blue bar, *n* = 8) (**D**). See the statistical table in Supplemental Table S1. One-way ANOVA followed by Tukey’s multiple comparisons test, ** *p* < 0.01, *** *p* < 0.001, n.s.—no significance.

Electrophysiology was employed to assess the functional myelination of the CC. The representative coronal brain sections used for these electrophysiological assessments are shown in Figure 7A. The measured parameters, including Peak 1 amplitude, Peak 2 amplitude, CV1, CV2, and the Peak 2/Peak 1 ratio, were analyzed separately using a one-way ANOVA followed by Tukey’s multiple comparisons test (Figure 7D; the statistical table is found in Supplemental Table S1). A unilateral HI injury led to a decrease in Peak 1 amplitude (myelinated axons) in the right/injured side of the CC (HI; *n* = 9, 0.36 ± 0.11 mV) compared to the left/uninjured control side (sham; *n* = 21, 0.88 ± 0.10 mV, one-way ANOVA, *p* = 0.0001, F (4, 49) = 39.60). In contrast, the amplitude of Peak 2 (non-myelinated axons) was not significantly altered (one-way ANOVA, *p* = 0.5824, F (4, 49) = 0.7200) (Figure 7C,D). Additionally, the conduction velocity of Peak 1 (CV1) was significantly attenuated in the right/injured side of the CC (HI; *n* = 9, 0.99 ± 0.02 m/s) compared to the left/control side of the CC (sham; *n* = 21, 1.09 ± 0.04 m/s, one-way ANOVA, *p* = 0.0001, F (4, 49) = 7.647). In contrast, the conduction velocity of Peak 2 (CV2) was not significantly altered after HI (one-way ANOVA, F (4, 49) = 2.136, *p* = 0.0904) (Figure 7C,D).

These results suggest that unilateral HI injury led to the degeneration of myelinated axons in the CC, which remained unmyelinated, while non-myelinated axons were not significantly affected. This conclusion is supported by the significantly elevated Peak 2/Peak 1 ratio amplitude in the right/injured side of the CC (HI; *n* = 9, 0.83 ± 0.14) compared to the left/control side of the CC (sham; *n* = 21, 0.40 ± 0.13, one-way ANOVA, *p* = 0.0001, F (4, 49) = 21.38) (Figure 7D).

The Peak 1 amplitude in the right/injured CC significantly improved after NPC (*n* = 8, 0.72 ± 0.13 mV, one-way ANOVA, vs. HI, *p* = 0.0001), CIMT (*n* = 8, 0.67 ± 0.09 mV, one-way ANOVA, vs. HI, *p* = 0.0001), and NPC + CIMT (*n* = 8, 0.82 ± 0.11 mV, one-way ANOVA, vs. HI, *p* = 0.0001) treatments and was significantly higher compared to the injured group (HI; *n* = 9, 0.36 ± 0.11 mV). The results indicated a significant difference in the Peak 1 amplitude after NPC (one-way ANOVA, vs. sham, *p* = 0.0061) and CIMT (0.67 ± 0.09 mV, one-way ANOVA, vs. sham, *p* = 0.0002) when compared to the sham animals (0.88 ± 0.10 mV). However, the difference between NPC + CIMT (one-way ANOVA, *p* = 0.6586) and the sham group was not significant, indicating full restoration (Figure 7D).

When compared with the sham animals (*n* = 21, 1.09 ± 0.04 m/s), the CV of Peak 1 (i.e., CV 1) was fully restored after NPC (*n* = 8, 1.05 ± 0.08 m/s, *p* = 0.3488) and NPC + CIMT (*n* = 8, 1.03 ± 0.06 m/s, *p* = 0.0541) treatments, but only partially restored after CIMT (*n* = 8, 1.01 ± 0.06 m/s, *p* = 0.0043) treatments (Figure 7D).

The Peak 2 amplitude, as well as the CV of Peak 2 (i.e., CV 2), was not affected by any treatment. However, the increased Peak 2/Peak 1 ratio observed following HI injury (*n* = 9, 0.83 ± 0.14) was normalized after NPC (*n* = 8, 0.42 ± 0.14, one-way ANOVA, *p* = 0.9956), CIMT (*n* = 8, 0.48 ± 0.10, one-way ANOVA, *p* = 0.5647), and NPC + CIMT (*n* = 8, 0.35 ± 0.12, one-way ANOVA, *p* = 0.8797) treatments when compared to the sham animals (0.40 ± 0.13) (Figure 7D).

Overall, the electrophysiology data suggest that CIMT treatment alone did not fully restore CV1 or Peak 1 amplitude, and NPC treatment alone was not sufficient to restore Peak 1 amplitude. However, all three treatments, especially NPC + CIMT, significantly protect myelinated axons and promote myelination of unmyelinated axons, particularly those with a larger diameter.

### 2.4. Transplanted NPC, CIMT, and NPC + CIMT Treatments Led to Functional Recovery

#### 2.4.1. Cylinder Test

**Figure 8:**
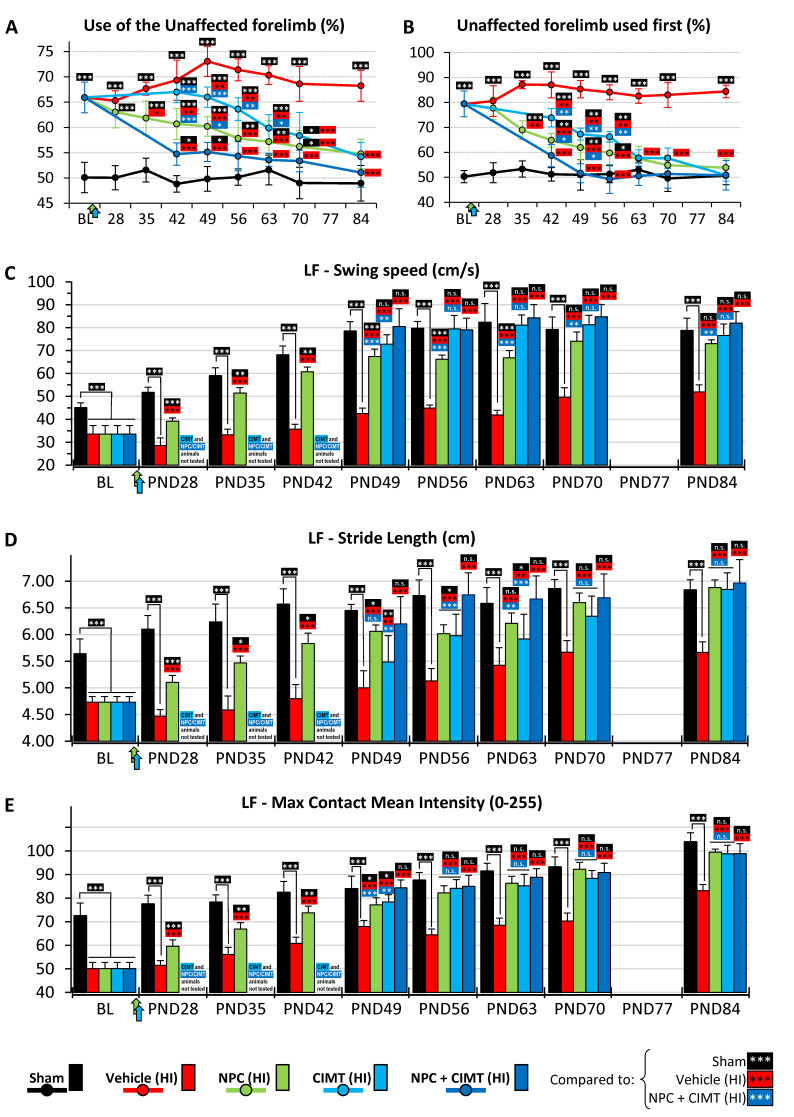
All treatments led to functional recovery. The cylinder test showed a clear preference for using the unaffected right forelimb in HI mice ((**A**), baseline time-point—BL). A progressive recovery was observed following all treatments when compared to the sham group (black line). The combinatorial treatment (NPC + CIMT; dark blue line) led to earlier and greater recovery than NPC transplantation (green line) or CIMT (blue line) alone (**A**). Similarly, the test also showed a strong preference for the HI mice to use the unaffected right forelimb to initiate a rearing sequence. Progressive recovery was observed following all treatments when compared to the sham group. NPC + CIMT led to earlier and enhanced recovery as compared to using NPC transplantation or CIMT alone (**B**). The CatWalk test demonstrated an impairment of HI animals for several parameters, including Swing Speed (**C**), Stride Length (**D**), and Paw Intensity (**E**) when compared to the sham mice (black bars) at baseline. A progressive improvement was observed after NPC (green bars), CIMT (blue bars), and NPC + CIMT (dark blue bars) treatments. NB: Following the onset of the treatments, recordings were not reliable for CIMT and NPC + CIMT animals for the first 3 time-points (i.e., PND28, 35, and 42) and were not reported. Furthermore, the PND77 time-point was skipped as the analysis of the partial data obtained at PND70 showed a “plateau” of the values, and consequently, a time-point after two weeks (PND84 following PND70) was sought. Green and blue arrows show the timing of, respectively, the NPC and the CIMT treatments. * *p* < 0.05, ** *p* < 0.01, *** *p* < 0.001, n.s.—no significance.

Only the ipsilateral/right brain hemisphere is lesioned in our unilateral HI injury model. Therefore, the injured mice will have a preference for the use of the right unaffected forelimb (Figure 8A,B).

NB: Due to the CIMT protocol (i.e., muscular paralysis of the ipsilateral uninjured forelimb), the cylinder test could not be performed successfully before PND42 (Figure 1D). Animals received CIMT from PND23 to 32, but the paralysis lasted until at least PND35, on which date the recordings were not consistent (data not included). Mice were successfully tested at PND42 (i.e., approximately 3 weeks after the start of the treatments).

Prior to treatments, the baseline assessment at PND20 showed that HI mice preferentially used the right unimpaired forelimb for support during rearing exploration sequences (65.93 ± 3.05%, two-way ANOVA, followed by Bonferroni’s test, F = 13.69) as compared to the control mice (50.10 ± 3.00%, *p* < 0.0001, t = 12.33). This preference for the right forelimb gradually disappeared after treatments. However, the recovery occurred quicker following the combined therapy (NPC + CIMT) compared to the NPC or CIMT treatment alone. For example, at PND42 (i.e., 3 weeks after treatments), the mice were using the right forelimb less frequently: NPC (60.69 ± 3.06%, *p* < 0.0001, t = 7.38), CIMT (67.01 ± 2.11%, *p* = 0.7033, t = 1.82), and NPC + CIMT (54.73 ± 2.21%, *p* < 0.0001, t = 11.65), when compared to the vehicle (69.34 ± 3.97%), while the sham control was 48.82 ± 1.64%. The combined therapy led to a better and quicker recovery (54.73 ± 2.21%), when compared to NPC (60.69 ± 3.06%, *p* < 0.0001, t = 5.23) or CIMT (67.01 ± 2.11%, *p* < 0.0001, t = 9.79) treatments alone. By the end of the period assessment (i.e., PND84, 9 weeks after treatments), there was a complete recovery—NPC (52.11 ± 2.58%, *p* = 0.0733, t = 2.70), CIMT (51.19 ± 3.07%, *p* = 0.8182, t = 1.75), and NPC + CIMT (51.07 ± 2.92%, *p* = 0.9180, t = 1.69)—compared to the sham group (48.95 ± 3.51%). The injured HI group maintained a strong preference for the right forelimb (68.23 ± 3.06%, *p* < 0.0001) until the end of the testing period (Figure 8A).

When we looked at which forelimb was used first to start a rearing sequence, we also found asymmetry. At baseline (PND20), the HI mice showed a clear preference for the right unimpaired forelimb (79.43 ± 5.15%, two-way ANOVA, followed by Bonferroni’s test, F = 22.79) as compared to the sham mice (50.28 ± 2.43%, *p* < 0.0001, t = 15.42). The preference for starting a rearing activity with the right forelimb gradually disappeared after treatments, but the recovery took place quicker after the combined therapy (NPC + CIMT) compared to NPC or CIMT treatment alone. Three weeks after treatments (PND42), the mice were starting their exploring sequence less frequently with the right forelimb: NPC (64.81 ± 2.72%, *p* < 0.0001, t = 12.89), CIMT (73.83 ± 3.67%, *p* < 0.0001, t = 6.99), and NPC + CIMT (58.68 ± 4.43%, *p* < 0.0001, t = 15.36), when compared to the vehicle (87.04 ± 5.23%), while the sham control was 51.20 ± 2.68%. The combined therapy led to better and quicker recovery (58.68 ± 4.43%), when compared to NPCs (64.81 ± 2.72%, *p* = 0.003, t = 3.65) or CIMT (73.83 ± 3.67%, *p* < 0.0001, t = 8.21) alone. At the end of the assessment period (i.e., 9 weeks after treatments), there was a full recovery—NPC (53.85 ± 3.61%, *p* < 0.0001, t = 17.71), CIMT (50.86 ± 2.35%, *p* < 0.0001, t = 17.75), and NPC + CIMT (50.80 ± 5.88%, *p* < 0.0001, t = 18.20)—compared to the sham group (50.65 ± 3.64%). The vehicle group maintained the preference for using the right forelimb (84.41 ± 2.53%) throughout the testing period (Figure 8B).

#### 2.4.2. CatWalk Gait Assessment

Any alterations to brain subcortical and spinal cord network structures, which mediate walking, are known to impact locomotor performance. Several gait parameters were assessed to determine functional recovery following treatments with NPCs, CIMT, and the combination of NPC + CIMT. Here, we present (i) Swing Speed (Figure 8C), which is the speed of the paw while not in contact with the floor; (ii) Stride Length (Figure 8D), which is the distance between successive points of contact of the same paw; and (iii) Paw Intensity (Figure 8E), reflecting how the injury affects weight bearing.

NB: Due to the CIMT protocol, the CatWalk test could not be performed successfully early after the treatment (Figure 1D). The induced paralysis made the mice reluctant to walk consistently on the glass corridor. Thus, they were tested again at PND49 (successfully).

At baseline (PND20), the left/injured forelimb (LF) showed a significant decrease (two-way ANOVA, followed by Bonferroni’s test) in Swing Speed (33.52 ± 3.71 cm/s, F (20, 285) = 45.60), Stride Length (4.73 ± 0.11 cm, F (20, 285) = 15.85), and Paw Intensity (50.12 ± 2.59 intensity level −0 to 255−, F (20, 285) = 27.69), compared to the left forelimb of the sham animals (Swing Speed: 45.09 ± 2.07 cm/s, *p* < 0.0001, t = 6.37; Stride Length: 5.64 ± 0.27 cm, *p* < 0.0001, t = 8.09; and Paw Intensity: 72.55 ± 5.37, *p* < 0.0001, t = 15.93) (Figure 8C–E).

##### Swing Speed

After NPC transplantation, we observed a small but significant recovery as early as 1 week later (PND28) as the Swing Speed increased to 39.14 ± 1.48 cm/s, as compared to the vehicle group (28.52 ± 3.39 cm/s, *p* < 0.0001, t = 6.73). For animals treated with CIMT alone or NPC + CIMT, we observed recovery as soon as consistent recordings were possible (i.e., PND49): when compared to the vehicle group (42.50 ± 2.38 cm/s), CIMT and NPC + CIMT corrected the Swing Speed, respectively, to 72.78 ± 4.14 cm/s (*p* < 0.0001, t = 15.08) and 80.48 ± 7.76 cm/s (*p* < 0.0001, t = 18.91), while NPC treatment also improved it (67.37 ± 3.30 cm/s, *p* < 0.0001, t = 12.38). At that time-point, the NPC + CIMT (80.48 ± 7.76 cm/s) triggered a better and quicker recovery than NPCs (67.37 ± 3.30 cm/s, *p* < 0.0001, t = 6.02) or CIMT (72.78 ± 4.14 cm/s, *p* = 0.0048, t = 3.54) alone.

At the end of the study (PND84), complete recovery was observed following all three treatments. When compared to the sham group (78.84 ± 5.27 cm/s), Swing Speed was completely restored after NPC (73.03 ± 1.66 cm/s, *p* = 0.1091, t = 2.56), CIMT (76.60 ± 4.97 cm/s, *p* > 0.9999, t = 0.99), and NPC + CIMT (82.03 ± 4.94 cm/s, *p* > 0.9999, t = 1.41) treatments.

##### Stride Length

One week after NPC treatment, Stride Length improved to 5.10 ± 0.13 cm, as compared to the vehicle (4.47 ± 0.12 cm, *p* < 0.0001, t = 7.01).

Animals treated with CIMT alone or NPC + CIMT were assessed at PND49. When compared to the vehicle group (5.00 ± 0.32 cm), all three treatments improved Stride Length: NPC (5.10 ± 0.13 cm, *p* < 0.0001, t = 8.53), CIMT (5.49 ± 0.49 cm, *p* = 0.0010, t = 3.94), and NPC + CIMT (6.20 ± 0.51 cm, *p* < 0.0001, t = 9.65). At that time-point, the NPC + CIMT (6.20 ± 0.51 cm) triggered a better and quicker recovery than CIMT (5.49 ± 0.49 cm, *p* < 0.0001, t = 5.64) alone, but not significantly compared to NPCs alone (6.06 ± 0.12 cm, *p* > 0.9999, t = 1.04).

At the end of the study (PND84), all three treatments produced complete recovery. When compared to the sham group (6.84 ± 0.19 cm), Stride Length was completely restored after NPC (6.88 ± 0.14 cm, *p* > 0.9999, t = 0.29), CIMT (6.84 ± 0.31 cm, *p* > 0.9999, t = 0.00), and NPC + CIMT (6.96 ± 0.44 cm, *p* > 0.9999, t = 0.86) treatment.

##### Paw Intensity

One week after NPC treatment, Paw Intensity increased to 59.54 ± 2.70, as compared to vehicle (51.47 ± 2.04, *p* < 0.0001, t = 7.48).

The first recordings of animals treated with CIMT alone or NPC + CIMT were carried out at PND49. When compared to the vehicle group (68.00 ± 2.49), all three treatments improved Paw Intensity: NPCs (77.14 ± 2.97, *p* < 0.0001, t = 5.88), CIMT (78.38 ± 3.09, *p* < 0.0001, t = 6.66), and NPCs + CIMT (84.42 ± 3.29, *p* < 0.0001, t = 10.56). At that time-point, the NPC + CIMT (84.42 ± 3.29) treatment triggered a better and quicker recovery than NPCs (77.14 ± 2.97, *p* = 0.0002, t = 4.32) or CIMT (78.38 ± 3.09, *p* = 0.0032, t = 3.59) alone.

At the end of the study (PND84), all three treatments produced full recovery. When compared to the sham group (103.84 ± 3.86), Paw Intensity was completely restored after NPC (99.46 ± 1.29, *p* = 0.1319, t = 2.49), CIMT (98.41 ± 3.57, *p* = 0.0449, t = 2.86), and NPC + CIMT (98.86 ± 4.20, *p* = 0.0490, t = 2.84) treatment.

## 3. Discussion

In this study, we adapted and optimized the HI model of hemiplegic perinatal brain injury, as delineated in a previous publication [30], to investigate the therapeutic efficacy of NPC transplantation in conjunction with CIMT for neural structure repair and neurological functionality improvement. Individually, both therapeutic approaches led to significant structural and functional repair. Furthermore, our findings revealed a synergistic interplay between NPCs and CIMT when administered simultaneously, offering promising avenues for translational neuroscience.

### 3.1. Transplanted NPCs Survive, Differentiate Morphologically, and Engraft in Brain Tissue

The fate of NPCs after transplantation in the CC aligns with our previous findings [30], wherein the major fraction retained their positivity for Nestin, a neural stem cell marker [34]. These NPCs extended abundant projections directed towards cerebral regions such as the cortex and hippocampus. Their morphological differentiation and local engraftment demonstrated connections formed with the adjacent tissue, possibly mediated through adhesion molecules, secretion of trophic factors, or electrophysiological interactions [35,36,37]. Notably, the association with CIMT appeared to exert minimal influence on either the survival rate (5.12% versus 4.34%) or the differentiation of transplanted NPCs.

### 3.2. NPC Transplantation Leads to Structural and Functional Improvement

The structural and functional recovery elicited by the transplanted NPCs is consistent with our previous research findings [30]. Notwithstanding the modest survival rate, these exogenous NPCs facilitated both structural and functional rejuvenation.

The exogenous NPCs led to significant restoration of most of the brain structures investigated. However, some of them were not fully normalized, as shown by the brain, hippocampus, and lateral ventricles sizes, as well as the CA1 and CA3 neuronal counts. However, the CC was completely repaired anatomically and showed an increased endogenous oligodendrocyte population (YFP negative) that went above control levels, supporting successful repair mechanisms.

Following HI, subsequent motor impairment is a disconnection of the cortex from the midbrain, brain stem, and spinal cord as a consequence of subcortical damage to descending fiber tracts and/or direct damage to the motor cortex [17,33,38,39]. The observed functional recovery can be attributed toremyelination of the CC’s compromised axons, a hypothesis supported by electrophysiological evidence. This myelination involved endogenous oligodendrocytes, attracted to the lesion site by Nestin+ exogenous NPCs. This mechanism aligns with previous studies highlighting endogenous progenitors as pivotal key players in myelin restoration post-demyelinating injuries [40,41,42,43]. Furthermore, NPCs are known to produce and secrete molecules, e.g., PDGFα, IGF-1, BDNF, and EPO, that are fundamental to oligodendrogenesis [43,44,45] and improve brain repair via immunomodulatory pathways [46,47,48].

Exogenous elements can recruit endogenous cells in the regenerative process pathways [45,49,50]. Therefore, the NPC effects observed here likely occurred through indirect mechanisms such as trophic and/or biobridge support [47,51,52,53].

### 3.3. CIMT Also Leads to Structural and Functional Improvement

Likewise, CIMT produced significant structural repairs in most brain structures evaluated. However, exceptions were noted in the neuronal population of the cortex (i.e., somatosensory and primary motor regions) and the CC volume, which did not attain normalization. Additionally, the oligodendrocyte population of the CC was restored beyond control levels. However, this restoration remained inferior to the one achieved post-NPC intervention (i.e., 123.09% versus 142.69%, in comparison to 55.97% for HI and the sham baseline of 100%). Furthermore, CIMT promoted myelination of the unmyelinated fibers of the CC, albeit to a lesser extent than NPC treatment, as evidenced by electrophysiological readings. This is shown by a reduced amplitude (0.67 versus 0.72 mV) and a slower conduction velocity (1.01 versus 1.05 m/s). CIMT motor enhancements were visible from PND42 (with the cylinder test) and PND49 (with the CatWalk test; the first consistent time-point) and demonstrated normalization of motor functions by PND84. All investigated parameters, with the exception of Swing Speed, revealed delayed recovery when compared to the NPC approach. However, this disparity was attenuated over time, converging to normalized metrics by PND84.

Our findings, which demonstrated the efficacy of CIMT in motor function improvement, are in line with other animal studies utilizing diverse injury models. The principal underlying mechanism was the circumvention of growth-inhibitory signaling [18,20,27,54,55,56].

In a mouse model of CP, CIMT has been proposed to foster the remodelling of neurons within the motor cortex, particularly targeting dendrites and axons at the neurofilament level. Proteomic changes in the motor cortex, as well as in the spinal cord, involved proteins related to synapse stability, neuronal and axonal development as well as maintenance, and myelin formation [54]. This remodelling in a hemiplegic HI model was achieved via the inhibition of the Nogo-A/Nogo receptor as well as the RhoA/Rho-Associated kinase signaling cascade and led to motor improvement, as shown by the Rotarod and Front-Limb Suspension tests [27] or Beam and Grip tests [55]. The downregulation of RhoA/ROCK2 modulated Sox2/Fyn transcription in the brain to induce maturation and differentiation of oligodendrocytes [56].

Similarly, in a rat model of stroke, CIMT resulted in the downregulation of this signaling pathway in the region of the lesioned cortex and the upregulation of markers such as growth-associated protein-43, synaptophysin, vGlut1, and postsynaptic density-95 in the denervated cervical spinal cord [18]. Plasticity of the corticospinal tract, associated with a declined ratio of Phosphorylated-JNK/Total-JNK [20] and a role of AMPA receptor-mediated transmission in upper limb motor function [23], has also been demonstrated. Furthermore, this downregulation has been correlated with axonal growth, neurogenesis, and angiogenesis in the subventricular zone [21], as well as dendrites and dendritic spine plasticity in the sensorimotor cortex, along with an increase in the expression of synaptic Glutamate Receptors 2 [21,22].

The signal disruption of the Nogo receptor, a myelin-inhibiting protein, demonstrated the pivotal role of myelin remodelling in motor recovery, with oligodendrocytes being key players. In a neonatal HI mouse model using Botox for CIMT, Adams et al. showed motor recovery and proposed an association between the increased presence of inflammatory cells, specifically microglia/macrophages, but not with NPC activation or recruitment to the lesion site [26]. However, our study demonstrated the central role of endogenous oligodendrocytes in structural and functional improvement. It should be noted that our CIMT protocol was significantly different than theirs, i.e., age of neonates (PND7 versus PND8), hypoxia duration (45 versus 60 min), scheduling of Botox injections (PND23, 26, 29, and 32 versus PND15 and 22), and the behavioural assessment endpoint (PND84 versus PND35). Remarkably, our protocol produced a longer paralysis phase. However, their findings also confirmed that standalone CIMT (i.e., without any specific enriched environment or additional physical training) can indeed lead to significant improvement after injury.

### 3.4. NPCs and CIMT in Combination Have a Synergistic Effect on Functional Recovery

Combining NPCs with CIMT to achieve enhanced therapeutic outcomes is a promising strategy. Many therapeutic strategies have been explored in neonatal HI research. Notably, the use of enriched environments [57,58], metformin [50], NPC transplantation [30], and neuromodulation [48] have all been associated with significant injury improvement. Furthermore, in mouse models with early HI brain injury, the combination of CIMT with an enriched environment produced motor enhancements that were attributed to neurogenesis [59]. Similarly, CIMT and electroacupuncture post-HI showed motor improvement and increased NeuN levels as well as reduced GFAP expression in the cerebral cortex [25].

Interestingly, the association of CIMT and NPC treatments did not lead to further substantial gain in terms of structural recovery when compared to separate approaches. Nevertheless, there was a synergistic increase in the endogenous oligodendrocyte population, i.e., 182.04%, in contrast to 142.69% (NPC) and 123.09% (CIMT) monotherapies, and 55.97% (HI), with control normalized to 100%. Electrophysiological assessments revealed that the combined therapies bolstered the CAP Peak 1 amplitude, corroborating its synergistic potential. Furthermore, neurobehavioural evaluations, such as the cylinder test, showed that the combined intervention led to significantly earlier and amplified motor improvement. For example, the rearing sequences were initiated 58.68% of the time with the unaffected forelimb, which was better than with standalone NPC or CIMT treatment (64.81% and 73.83%) or without any treatment at all (HI, 87.04%), while a sham animals’ rate was 51.20%. This synergistic effect faded with time, and at the end of the study, all treated groups displayed control-level values. Similarly, the CatWalk test showed a synergistic effect on gait at PND49, which was the first measurable time-point.

Our prior research has shown that NPC treatment mainly relied on the recruitment of endogenous oligodendrocytes, mediated through a trophic effect [30]. Comparable cellular recruitment was also observed in the CC following CIMT treatment. However, an alternative mechanistic pathway is likely to be involved. Plasticity-based myelination, suppression of growth signal inhibition [27], angiogenesis, and neurogenesis [18,21,27] could explain the synergistic effect on oligodendrocyte recruitment and functional recovery when both CIMT and NPC treatment were applied.

In conclusion, the combination of regenerative (NPC transplantation) and rehabilitative (CIMT) strategies produced synergistic enhancements in functional recovery, characterized by earlier and more pronounced improvements. This supports the principle of a multimodal therapeutic approach for neonatal stroke and CP. This study further clarifies the intrinsic mechanisms of NPC transplantation in the CC and of CIMT. We demonstrated, for the first time, a promising synergistic effect of these two translationally relevant therapeutic strategies for the potential treatment of hemiplegic CP.

## 4. Materials and Methods

### 4.1. Animal Use

The experimental procedures as well as animal use and care were approved by the Animal Care Committee and Research Ethics Board at the University Health Network in accordance with the policies and procedures outlined by the Canadian Council of Animal Care. C57Bl/6 mice were housed under controlled conditions (12 h light/dark cycles, +24 °C temperature, and fed ad libitum with automatic watering). The day of birth was defined as postnatal day (PND) 0.

### 4.2. Study Design

At PND7, male and female mice were subject to either a sham surgery (“sham”; dissection but no carotid cauterization) or hypoxia-ischemia surgery (“HI”; permanent cauterization of the right common carotid). Following surgery, all mice were exposed to hypoxic conditions to trigger unilateral brain damage (Figure 1A). Mice were randomly and blindly selected from several litters and assigned to 5 experimental groups: “sham” was the control group and “HI + vehicle” included the injured animals. Following HI, the 3 other groups received a cellular treatment (i.e., NPCs transplanted in the CC), a rehabilitation therapy (i.e., CIMT), or a combination of both (Figure 1B). These groups were “HI + NPC”, “HI + CIMT”, and “HI + NPC + CIMT”. A number of outcome measures were assessed, including immunostaining and histology, electrophysiology, and behavioural testing (cylinder rearing test and the CatWalk test). Figure 1C shows a timeline of the project and Figure 1D clarifies the time-points of the study. All outcomes were assessed in a blinded fashion.

### 4.3. Hypoxia-Ischemia Model of Hemiplegic Perinatal Brain Injury

In this study, we adapted the experimental conditions of the Rice–Vannucci model of hypoxia-ischemia [31,60,61,62,63]. This model is known to generate variable levels of unilateral brain injury, described as mild, moderate, or severe. We standardized the surgical procedure and reduced the hypoxia time to obtain a consistent mild HI injury as detailed in our previous publication, i.e., 8% oxygen for 45 min [30]. We ensured that the brain lesions triggered motor impairments, while avoiding complete destruction of the brain structures. Figure 1A describes the HI surgery, which is detailed in our previous publication [30].

We used PND7 pups based on histological similarities with human fetuses at 32–34 weeks of gestation [64,65]. The death rate was 9–10% after the hypoxia procedure. Animals (<2%) with a moderate or severe injury (no visible CC; >30% liquefied cavity) were excluded from the study.

### 4.4. Adult NPC Transplantation

Adult NPCs were obtained from the subventricular zone of transgenic adult mice expressing yellow fluorescent protein (YFP) [strain 129-Tg (ACTB-EYFP) 2 Nagy/J; The Jackson Laboratory, Bar Harbor, ME, USA] according to a previously described protocol [66]. NPCs were transplanted in the CC at PND21, the age corresponding to a 2-year-old child from a developmental perspective [67], as CP is often not formally diagnosed until age 2 or 3 (Figure 1B–D and Figure 2). This protocol has been previously described [30]. Using the Bregma point as a reference, 2.5 μL of NPC suspension (50,000 cells/μL) was injected at 2 locations: on the right (ipsilateral to injury) side (+1 mm lateral/rostral and +1 mm lateral/caudal), which targets the CC (anterior site 1 mm depth; posterior site 1 mm depth/10° angle) at a rate of 250 nL/min. Each mouse was given 0.1 mL of 0.9% saline solution, 0.1 mL of Buprenorphine (0.048 mg/kg, CMARC, McGill university, Montreal, QC, Canada), and Sandimmune (10 mg/kg, Sandimmune^®^I.V., Cyclosporine concentrate, 50 mg/mL, Novartis, Toronto, ON, Canada).

The pups were weaned, and food supplement was added to the cage (Kitten Milk Replacer; PetAg, Hampshire, IL, USA). Mice were given Buprenorphine and Sandimmune twice daily for the following 2 days. Afterwards, mice were given Cyclosporine in drinking water (0.08 mg/mL; 20 mg/mL solution, Chiron Compounding Pharmacy, Guelph, ON, Canada) and were frequently monitored.

### 4.5. Constraint-Induced Movement Therapy (CIMT)

We modelled CIMT using intramuscular injections of Botulinum toxin (BOTOX, Allergan), targeting the biceps, the triceps, and the supraspinatus muscle of the unaffected forelimb (Figure 1B–D). For a final working solution of 0.05 units/mL, 50 units were reconstituted in 1 mL of phosphate buffer saline. At PND23, under standard isoflurane anesthesia (2.5%), 3 µL of BOTOX solution was manually injected into each muscle using a 10 μL microsyringe (Microliter^TM^ Syringe, Hamilton^®^, Reno, NV, USA). The animals were monitored for 15 min post-recovery and twice daily to ensure effective forelimb paralysis. As the paralysis weakened after 72 h, 3 other sets of injections were performed every 3 days (i.e., PND26, 29, and 32), allowing for consistent immobilization of the forelimb for 12 days (from approximately PND23 to 35). Animals receiving botox injections, as well as control animals (vehicle, PBS), were provided soft food (i.e., food pellets imbibed with drinking water) and rehydrated milk powder in small petri dishes placed on the cage floor to facilitate their independent feeding despite the paralysis. These supplements were changed at least 2 times a day. This paralysis prevented accurate neurobehavioural testing before PND42 (cylinder test) and PND49 (CatWalk test). Supplemental Figure S1 shows the body weight (BW) of treated and vehicle control animals during the CIMT treatment period, i.e., PND23 to 35 and confirm their normal BW and BW gain. A repeated measures ANOVA was performed and showed transient differences in BW between groups in males (F (1.161, 13.93) = 4.793 with a *p* value of 0.0417) and females (F (1.294, 15.52) = 5.751 with a *p* value of 0.0229). While slight individual variations were observed, they were considered non-adverse as they were transient and were fully resolved at PND35 with no other correlated adverse signs.

### 4.6. Structural Assessment

#### 4.6.1. Tissue Preparation

Following the last neurobehavioural testing at PND84 (“Week 12”, or “9 weeks after NPC transplantation”, or “7 weeks after the end of CIMT treatment”; Figure 1D), animals were deeply anesthetized and transcardially perfused with 0.1 M PBS followed by 4% paraformaldehyde/0.1 M PBS, pH 7.4. Brains were then extracted, post-fixed for 4 h, cryoprotected, embedded, and frozen, as previously described [68]. Then, 20 μm coronal sections from Bregma −2.5 to +1.2 mm were collected on Superfrost+ Slides (Fisher Scientific, Ottawa, ON, Canada) on the cryostat and stored at −80 °C for later use.

#### 4.6.2. Immunostaining and Histology

Frozen sections were thawed in double-distilled water, spread using fine brushes, and air-dried before being rehydrated in PBS. For immunostaining, a blocking step was performed for 1 h at room temperature (RT) in a solution of 2% fetal bovine serum and 0.1% TritonX-100 in 0.1 M PBS. The primary antibodies used were mouse anti-GFAP (MAB3402; 1:1000; Millipore-Sigma, Oakville, ON, Canada) for astrocytes; rabbit anti-Olig2 (1:400; Ab9610; Millipore-Sigma, Oakville, ON, Canada) for oligodendrocytes; mouse anti-APC (CC1; 1:250; Ab16794; Toronto, ON, Canada) for mature oligodendrocytes and astrocytes; Cy3-conjugated mouse anti-Nestin (1:500; MAB353C3; Millipore-Sigma, Oakville, ON, Canada) as an NPC marker; and mouse anti-NeuN (1:500; MAB377; Millipore-Sigma, Oakville, ON, Canada) for neurons. Double-staining was performed using 2 primary antibodies with no cross-reactivity. The brain sections were incubated overnight at +4 °C with the primary antibodies diluted in the blocking solution. The following day, the sections were washed three times in 0.1 M PBS (washes of 5, 10 and 15 min at RT) and incubated with suitable secondary antibodies (1:400 in 0.1 M PBS for 1 h at RT): Alexa Fluor 568 IgG (anti-rabbit A11011; anti-mouse A11031; Life technologies, Burlington, ON, Canada) and Alexa Fluor 647 IgG (anti-mouse A21235; anti-rabbit A21244; Life technologies, Burlington, ON, Canada). DAPI (1:1000; D3571; Life technologies, Burlington, ON, Canada) was also used to stain the A-T rich DNA regions. The sections were then washed three times in 0.1 M PBS. Slides were mounted in Mowiol (Calbiochem; Millipore-Sigma, Oakville, ON, Canada), covered with coverslips, and kept at 4 °C. As a negative control for each secondary antibody, we performed the immunofluorescence protocol without primary antibodies. We used the following strategies to reduce nonspecific binding of the primary antibody: blocking step with 2% fetal bovine serum, dilution of antibody in the blocking solution, and multiple washes in PBS. Employed in these experimental conditions, these primary antibodies were considered to be specific with limited nonspecific binding [30,48,68].

For histological analysis, Luxol Fast Blue (LFB) and Hematoxylin and Eosin (H&E) staining were performed as previously described [69].

#### 4.6.3. Quantification

Cell counting and quantitative neuroanatomical assessments were performed in a blinded fashion. The following numbers of mice were included in the quantification: 10 (sham), 15 (HI + vehicle), 15 (HI + NPC), 15 (HI + CIMT), and 15 (HI + NPC + CIMT). Stereological assessments were performed on various brain structures.

The CC area and volume were evaluated using a Cavalieri estimator (Stereo Investigator^®^, MBF Bioscience, Williston, VT, USA) on the epifluorescence microscope. Using Stereo Investigator^®^, the contour of the CC was traced manually on the computer screen at low microscope magnification. Then, at higher magnification, each YFP+ cell (visible under fluorescence) was tagged on the computer monitor by a virtual marker (e.g., +, o, x). Using the motorized platform to move around, the whole CC area that was delineated was searched and marked for YFP+ cells on an optical section corresponding to the middle of the 20 μm physical section. The software displayed the total number of markers used, i.e., the number of NPCs, for each section. This procedure was repeated on all of the physical serial sections of interest covering both anterior and posterior transplantation sites and the surrounding areas (i.e., 2 × 5 brain sections) that were included in a coronal region of 3.2 mm thickness (Bregma −2 to +1.2 mm). The survival rate in animal A (SR_A_) was

SR_A_ = ∑(estimated cell number in 5 anterior + 5 posterior sections)/250,000 × 100

The survival rate of each group was therefore the mean of the individual rates:

$${\mathrm{SR}}_{\mathrm{Group}}=\overset{-}{x}({\mathrm{SR}}_{A}+{\mathrm{SR}}_{B}+{\mathrm{SR}}_{C}+\ldots )$$

To characterize the fate of the transplanted NPCs, YFP+ cells were also checked for double staining for NeuN (neurons), Olig2 (oligodendrocytes), or Nestin (neural stem cells) markers. Cells positive for these markers were also tagged on the computer using the same approach that was used for YFP+ cells, and double-stained cells were counted. Regarding the GFAP marker (astrocytes), to confirm a differentiation from NPC to astrocytes, meticulous assessments were performed to locate potential cell bodies and extensions simultaneously positive for GFAP and YFP. Since the goal was to characterize the transplanted cells, these evaluations were restricted to the area where YFP+ cells were observed and did not extend to the whole CC. Approximately, 2 × 5 coronal sections per animal over the 2 transplantation sites (±0.2 mm around Bregma −1 mm and Bregma +1 mm, i.e., sections every 4th slide). The numbers obtained in one animal from 2 × 5 physical sections for a specific cell type were added together and then divided by the total number of YFP+ cells counted in that animal, which produced the rate of differentiation in that type of cell. Individual values were subsequently used to obtain an average differentiation rate per treatment group.

The same counting method was used for Olig2+ cells that were counted within the delineated CC (i.e., about 24 sections, taken every 6th section). Regarding the mature (Olig2+/CC1+) and immature (Olig2+/CC1−) oligodendrocytes in the CC, they were counted in 3 pictures taken on a confocal microscope (magnification ×20) from similar positions on each coronal section, covering both transplantation sites (±1.2 mm rostro-caudally from Bregma, 12–15 sections taken every 10th to 8th section).

The neuronal population (NeuN+) was also counted in the hippocampus (CA1 and CA3) as well as in the primary motor cortex (PMC) and somatosensory cortex (SSC) based on representative pictures. These pictures were taken at similar locations across all brain sections based on anatomical landmarks such as the edge of the section, the midline, or the shape of the hippocampus. Nine to twelve coronal sections per animal (±1.2 mm rostro-caudally from Bregma, taken approximately every 10th section). The thickness (y-plane) of the PMC and SSC were also assessed.

The lateral ventricles and hippocampus were delineated, and their areas were estimated using the Stereo Investigator^®^ on 15 coronal LFB/H&E brain sections per animal taken every 4th section. These sections covered approximately a region from Bregma −0.2 to +1.2 mm. Similarly, the whole brain as well as each hemisphere area were evaluated on LFB/H&E sections that covered the Bregma −2 to +1.2 mm region. These LFB/H&E sections, when appropriate, were also used to obtain additional data for cortex thickness and CC area. Anatomical landmarks (e.g., the CC, the anterior commissure, the lateral ventricle, the caudate, or the hippocampus) were used to finely harmonize and maintain consistency between coronal sections and to validate comparisons between the animals and groups.

The raw numbers of cells in a specific brain structure vary according to the rostro-caudal position of the brain section, causing high disparity levels within one animal. To circumvent this problem, we calculated for each brain section the percentage of cells in the right versus left brain structure compared to the total number of cells within this structure. We then performed an average that was associated with one animal. We subsequently calculated the mean by treatment group. For the areas, we had a similar approach. This approach allowed for normalization between brain regions (e.g., transplantation sites at Bregma −1 mm and +1 mm) and animals.

### 4.7. Functional Assessment: Electrophysiology

Before decapitation and dissection of the brain, mice were deeply anesthetized with sodium pentobarbital (60 mg/kg, i.p.) and underwent trans-cardiac infusion with cold 95% O_2_/5% CO_2_ saturated sucrose-substituted artificial cerebrospinal fluid (aCSF). aCSF contained (in mM) sucrose, 210; NaHCO_3_, 26; KCl, 2.5; CaCl_2_, 1; MgCl_2_, 4; NaH_2_PO_4_, 1.25; and D-glucose, 10. Eight serial 400μm thick coronal slices of the brain containing the CC were obtained on Leica vibrating microtome VT1200S (Concord, ON, Canada) (Figure 7A) [70]. Isolated CCs were severed at the mid-point and separated into left and right halves. The slices were incubated in identifiable positions on a mesh in oxygenated aCSF containing (in mM) NaCl, 125; NaHCO_3_, 26; KCl, 2.5; CaCl_2_, 2; MgSO_4_, 1.3; NaH_2_PO_4_, 1.25; and D-glucose, 10, at RT with 95%O_2_-5%CO_2_ for at least 1 h before electrophysiological recording.

Compound action potentials (CAPs) were acquired using suction electrodes for both stimulating and recording, as previously described [70]. CAPs recorded from the CC provided information regarding myelinated axons, as demonstrated by the fast conduction velocity (CV) first peak (Peak 1). In contrast, non-myelinated axons were assessed using the CV second peak (Peak 2). The recordings were performed in a 0.5 mL bath with a 2.8 mm distance between two electrodes continuously perfused at 1 mL/min with aCSF oxygenated by 95% O_2_/5% CO_2_ (Figure 7B). The stimulating pulses of 0.1 ms duration and varying amplitude (0.01~1.5 mA) were applied via the PSIU6 stimulus isolation unit Grass S88 dual-channel stimulator (Grass Technologies, West Warwick, RI, USA). Direct current CAPs were recorded with an Axoprobe 1A amplifier (Axon Instruments/Molecular devices, San Jose, CA, USA). The signals were processed using pClamp version 8 software and DigiData 1320A using a 100 kHz low-pass filter (Axon Instruments/Molecular Devices, San Jose, CA, USA). Data analysis was performed offline using Clampfit10 after filtering the signals with a 10 kHz low-pass filter.

The following number of mice were included in this experiment: 10 (sham), 9 (HI + vehicle), 8 (HI + NPC), 8 (HI + CIMT), and 8 (HI + NPC + CIMT).

### 4.8. Functional Assessment: Neurobehavioural Testing

In total, 10 (sham), 15 (HI + vehicle), 15 (HI + NPC), 15 (HI + CIMT), and 15 (HI + NPC + CIMT) mice completed the functional tests detailed below. The researchers who performed the functional assessments were blinded to the identity of the mice. The assessments started on PND20/21 (baseline) and then were performed on a weekly basis until PND84. Due to the CIMT procedures, the second time-point had to be delayed until the mice could produce consistent neurobehavioural measures, i.e., PND42 (cylinder test) and PND49 (CatWalk test). Furthermore, the PND77 time-point was skipped as the analysis of the partial data obtained at PND70 showed a “plateau” of the values between the treated groups and the control. We therefore estimated that a time-point after two weeks (PND84 following PND70) was acceptable.

#### 4.8.1. The Cylinder Test

The cylinder test was used to assess preferential forelimb use. Each mouse was placed in a clear plastic cylinder, and its rearing activity was recorded for 5 consecutive minutes with a camera placed beneath the apparatus. The placement of the whole palm on the cylinder wall demonstrated the use of that forelimb for body support. The use of the right (R) unaffected versus left (L) affected forelimb was calculated as a percentage of total contacts: R/(R + L) × 100. Additionally, the rate of first forelimb use in a rearing sequence was calculated. 

#### 4.8.2. The CatWalk Gait Test

The CatWalk^TM^XT (Noldus Information Technology; Leesburg, VA, USA) was employed to measure walking performance. Each animal walked freely through a corridor on a glass walkway illuminated with beams of light from below. A successful walking trial was defined as having the animal walk at a steady speed (no stopping, rearing, or grooming), and three to five successful trials were collected per animal. The footprints were recorded using a camera positioned below the walkway, and footprint classification was manually corrected to ensure accurate readings [71]. CatWalk^TM^XT software (v10.6) was then used to analyze several gait parameters including Swing Speed (cm/s), Stride Length (cm), and Paw Intensity (arbitrary units, 0–255).

### 4.9. Statistical Analysis

Sample sizes were calculated to have a 90% chance of detecting effects at an alpha level of 0.05, using prior data from our laboratory [30]. For quantification, the value of the left contralateral side was normalized to 100%. All statistical analyses were performed using GraphPad Prism (v6.01; Boston, MA, USA). A D’Agostino and Pearson omnibus normality test was performed. For cell survival, an unpaired *t*-test was used. For NPC differentiation a one-way ANOVA test was used.

For electrophysiology, when the data met the normality and homoscedasticity assumptions, we used a one-way ANOVA followed by Tukey’s multiple comparisons test for all measured parameters, including Peak 1 amplitude, Peak 2 amplitude, CV1, CV2, and the Peak 2/Peak 1 ratio separately.

For other comparisons, we used a two-way ANOVA, followed by Bonferroni post hoc test. F values; exact t values; and, when available, exact *p* values were reported. The values reported are mean ± standard deviation (SD). n.s. = not significant; * *p* < 0.05; ** *p* < 0.01; *** *p* < 0.001.

## Data Availability

All inquiries should be directed to the corresponding author. The data are available upon reasonable request.

## Supplementary Materials

The following supporting information can be downloaded at https://www.mdpi.com/article/10.3390/ijms25179403/s1.
