# Supplementary material for: Constraint-Induced Movement Therapy (CIMT) and Neural Precursor Cell (NPC) Transplantation Synergistically Promote Anatomical and Functional Recovery in a Hypoxic-Ischemic Mouse Model
Source: Int J Mol Sci. 2024 Aug 29;25(17):9403. doi: 10.3390/ijms25179403 (PMC11395467; doi:10.3390/ijms25179403)
Supplement: Supplementary file 1 [file ijms-25-09403-s001.zip › ijms-3033214-supplementary.pdf]

Supplemental Material

Supplemental Table S1. Statistical table of electrophysiology data referring to Figure 7D

Parameters	Group size	Test	Statistic	P values	Statistic 2	P values 2
Peak 1(mV)	Sham n=21<br>HI n=9<br>NPC n=8<br>CIMT n=8<br>NPC+CIMT n=8	One-Way ANOVA<br>Tukey's multiple comparisons test	F (4, 49) = 39.60	P=0.0001	Sham vs. HI<br>Sham vs. NPC<br>Sham vs. CIMT<br>Sham vs. NPC + CIMT<br>HI vs. NPC<br>HI vs. CIMT<br>HI vs. NPC + CIMT<br>NPC vs. CIMT<br>NPC vs. NPC + CIMT<br>CIMT vs. NPC + CIMT	0.0001<br>0.0061<br>0.0002<br>0.6586<br>0.0001<br>0.0001<br>0.0001<br>0.8805<br>0.3432<br>0.0521
Peak 2(mV)	Sham n=21<br>HI n=9<br>NPC n=8<br>CIMT n=8<br>NPC+CIMT n=8	One-Way ANOVA<br>Tukey's multiple comparisons test	F (4, 49) = 0.7200	P=0.5824		
CV1 (m/s)	Sham n=21<br>HI n=9<br>NPC n=8<br>CIMT n=8<br>NPC+CIMT n=8	One-Way ANOVA<br>Tukey's multiple comparisons test	F (4, 49) = 7.647	P=0.0001	Sham vs. HI<br>Sham vs. NPC<br>Sham vs. CIMT<br>Sham vs. NPC + CIMT<br>HI vs. NPC<br>HI vs. CIMT<br>HI vs. NPC + CIMT<br>NPC vs. CIMT<br>NPC vs. NPC + CIMT<br>CIMT vs. NPC + CIMT	0.0001<br>0.3488<br>0.0043<br>0.0541<br>0.1343<br>0.9300<br>0.5072<br>0.5354<br>0.9365<br>0.9365
CV2 (m/s)	Sham n=21<br>HI n=9<br>NPC n=8<br>CIMT n=8<br>NPC+CIMT n=8	One-Way ANOVA<br>Tukey's multiple comparisons test	F (4, 49) = 2.136	P=0.0904		
Peak2/Peak1	Sham n=21<br>HI n=9<br>NPC n=8<br>CIMT n=8<br>NPC+CIMT n=8	One-Way ANOVA<br>Tukey's multiple comparisons test	F (4, 49) = 21.38	P=0.0001	Sham vs. HI<br>Sham vs. NPC<br>Sham vs. CIMT<br>Sham vs. NPC + CIMT<br>HI vs. NPC<br>HI vs. CIMT<br>HI vs. NPC + CIMT<br>NPC vs. CIMT<br>NPC vs. NPC + CIMT<br>CIMT vs. NPC + CIMT	0.0001<br>0.9956<br>0.5647<br>0.8797<br>0.0001<br>0.0001<br>0.0001<br>0.8808<br>0.8089<br>0.2668

Supplemental Figure S1

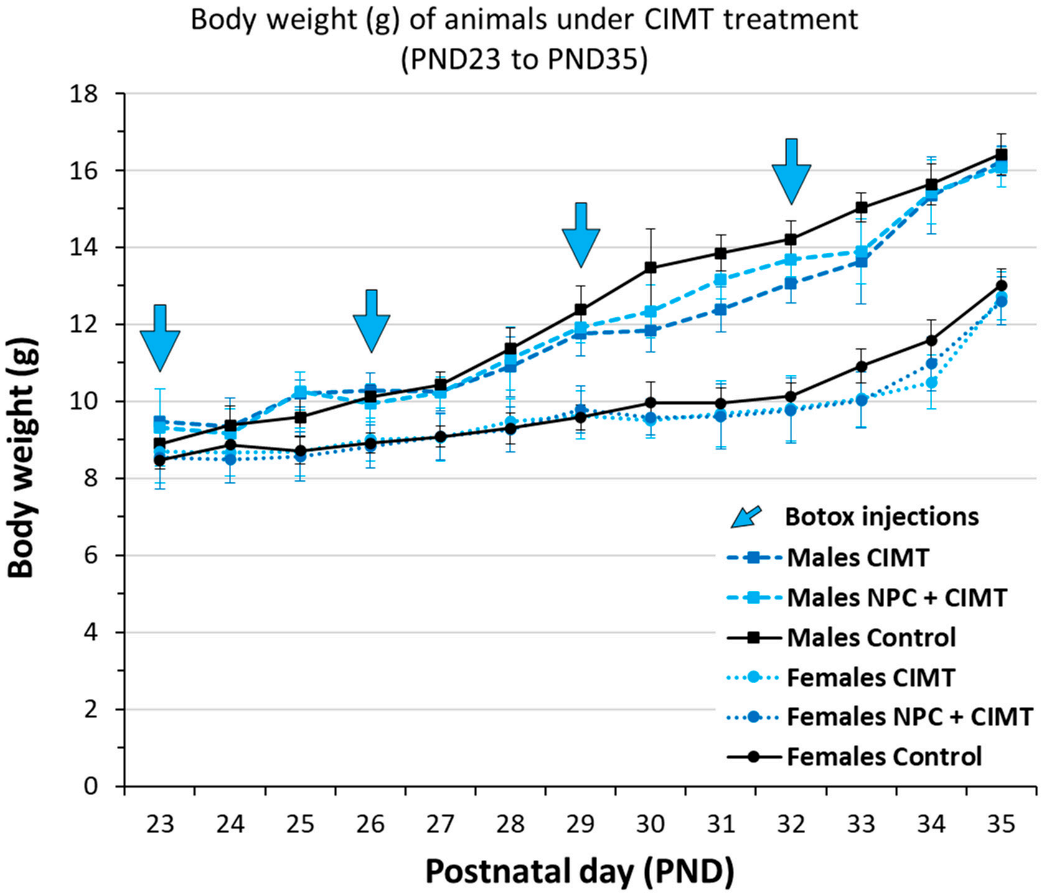

			PND	23	24	25	26	27	28	29	30	31	32	33	34	35
Males	CIMT	*n=6*	***Mean***	**9.46**	**9.34**	**10.21**	**10.29**	**10.25**	**10.90**	**11.76**	**11.85**	**12.39**	**13.05**	**13.64**	**15.34**	**16.24**
			SD	0.85	0.76	0.35	0.45	0.51	0.78	0.57	0.57	0.58	0.50	1.11	1.00	0.39
	NPC + CIMT	*n=8*	***Mean***	**9.31**	**9.18**	**10.26**	**9.95**	**10.23**	**11.11**	**11.91**	**12.33**	**13.18**	**13.69**	**13.89**	**15.44**	**16.09**
			SD	1.00	0.63	0.49	0.49	0.40	0.82	0.39	0.69	0.51	0.46	0.85	0.83	0.51
	Control	*n=8*	***Mean***	**8.90**	**9.38**	**9.58**	**10.12**	**10.43**	**11.37**	**12.38**	**13.47**	**13.85**	**14.22**	**15.03**	**15.63**	**16.42**
			SD	0.35	0.48	0.48	0.30	0.33	0.55	0.61	1.02	0.46	0.47	0.37	0.53	0.53

F (1.161, 13.93) = 4.793 with p = 0.0417

Females	CIMT	*n=6*	***Mean***	**8.69**	**8.66**	**8.69**	**9.01**	**9.06**	**9.49**	**9.64**	**9.51**	**9.67**	**9.81**	**10.07**	**10.50**	**12.73**
			SD	0.60	0.59	0.59	0.56	0.60	0.91	0.73	0.82	0.81	0.97	0.84	1.04	1.12
	NPC + CIMT	*n=7*	***Mean***	**8.53**	**8.49**	**8.57**	**8.83**	**9.09**	**9.27**	**9.79**	**9.59**	**9.60**	**9.76**	**10.03**	**10.97**	**12.60**
			SD	0.81	0.60	0.63	0.56	0.62	0.59	0.62	0.47	0.85	0.85	0.73	0.70	0.62
	Control	*n=7*	***Mean***	**8.47**	**8.87**	**8.72**	**8.92**	**9.08**	**9.30**	**9.58**	**9.97**	**9.95**	**10.13**	**10.92**	**11.58**	**13.02**
			SD	0.23	0.33	0.35	0.25	0.26	0.40	0.33	0.54	0.40	0.34	0.45	0.54	0.41

F (1.294, 15.52) = 5.751 with p = 0.0229

Supplemental Figure S1. Body weight records during CIMT treatment. Animals were weighed from postnatal day (PND) 23 to 35. Botox injections occurred on PND23, 26, 29 and 32. On PND35, the paralysis due to the toxin was completely gone and the animals fully recovered. A repeated measures ANOVA was performed and differences between groups were observed (PND 30-33) but were not detected at PND35.
